# Ethno-pharmacological investigations of *Moringa stenopetala* Bak. Cuf. and its production challenges in southern Ethiopia

**DOI:** 10.1371/journal.pone.0274678

**Published:** 2022-09-23

**Authors:** Azene Tesfaye, Agena Anjulo, Addisu Fekadu, Kassaw Beyene, Abebe Girma, Birhanu Gemeda, Gebremaryam Temesgen, Fistum Wolde, Eyob Mulugeta, Aseer Manilal

**Affiliations:** 1 College of Medicine and Health Sciences, Arba Minch University, Arba Minch, Ethiopia; 2 Ethiopian Forest Development, Addis Ababa, Ethiopia; 3 Department of Biology, Arba Minch University, Arba Minch, Ethiopia; 4 Department of Biology, Woldia University, Woldia, Ethiopia; 5 Biodiversity Research and Conservation Center, Arba Minch University, Arba Minch, Ethiopia; 6 Department of Chemistry, Arba Minch University, Arba Minch, Ethiopia; Bangladesh Agricultural University, BANGLADESH

## Abstract

**Introduction:**

*Moringa stenopetala* Bak. Cuf. is a native plant of Ethiopia with important nutraceutical applications. However, little is known about its nutritional, ethno-pharmaceutical and therapeutic properties. Hence, the present study sought to assess the nutraceutical applications of *M*. *stenopetala* among traditional healers in southern Ethiopia.

**Methods:**

A community-based cross-sectional study was conducted on 50 selected administrative units in Gamo Gofa, Segen areas and south Omo zones of southern Ethiopia from May to June 2020. Data were gathered using a semi-structured interview, field observation, and group discussion. Both quantitative and qualitative data were analysed using Excel 2019 and open code version 4.03, respectively. The results were presented using descriptive statistics, with the fidelity level (FL)% used to distinguish the preferential use of various plant parts.

**Results:**

A total of 120 individuals participated in the study, and the majority of them, 89 (74.2%), were male and farmers by occupation. Eight four (70%) of them were residents of the Gamo Gofa Zone. The fidelity level revealed that the leaf and root were the most commonly used parts for nutraceutical purposes. Remarkably, *M*. *stenopetala* is used to treat human ailments such as leprosy and kidney and liver infections via various modes of utilisation and administration. As a result, the most common methods of utilising plant products are chewing or consuming crushed plant parts, and the oral route is the much-preferred method of application. On the other hand, the larvae of Moringa moth *Nurda blitealis*, are a defoliating insect during the rainy season and have been identified as a limiting factor for its production.

**Conclusions:**

The nutraceutical aspects of *M*. *stenopetala* are extremely important to the rural community in southern Ethiopia. However, the defoliating moth larvae threaten its growth and biomass production, necessitating the need to manage and improve the plant’s productivity and sustainable use. Additionally, conducting experimental studies to validate the plant’s pharmacological potential correspond to a milestone in drug discovery.

## Introduction

*Moringa stenopetala* (Bak. Cufod) belonging to the family Moringaceae, is native to arid and semi-arid regions of Africa [1, 2]. It is mainly distributed in southern Ethiopia, eastern Kenya, and central Somalia [2–4]. The plant is widely grown in southwest Ethiopia, between 350 and 1850 meters above sea level, in terraced hilly cropped fields and home gardens, mainly in the lowlands of Gamo, Gofa, Segen, Wolaita and south Omo zones [5, 6].

A review of the literature showed that *M*.*stenopetala* is primarily cultivated for nutraceutical purposes [1, 7]. However, millions in the region rely on the plant for their daily needs. For example, it is used as a leafy vegetable in a well-known traditional dish [8], as well as cooked and eaten like cabbage, hence the name "cabbage tree" [9]. Moreover, it has remarkable medicinal values, and different parts are effective against various human ailments. For instance, it is widely used to treat wounds [9], diarrhoea, [5] vomiting, digestive problems, blood pressure [6], diabetes, rheumatism, cold problems, goitre, liver, and pancreatic diseases, malaria, high blood pressure, asthma, diabetes, stomach pain, and the removal of the retained placenta [10]. It also has anti-cancer properties and can treat various cancers in various organs [3, 11]. The root cortex, in particular, is used to stimulate appetite [12] and combat abdominal constipation [13], bronchitis and fever. In addition, the stem bark is used to treat eye disorders and to detoxify or eliminate the toxicity of venomous bites [6].

Interestingly, the plant is naturally drought-resistant and can survive in harsh environments, so it is a source of nutrition and medication during the dry season. Albeit the plant plays a significant role in everyone’s life in the community; however, its nutritional and therapeutic benefits have not been examined, and it is considered an underutilised plant [5, 6].

Exploring nutritional uses, therapeutic benefits, and growth challenges is a great way to validate its use by traditional healers. However, it is also necessary to provide information about the parts used, the ailment addressed, the mode of drug preparation and the method of consumption. Therefore, it emphasises the importance of ethnopharmacological research into nutritional therapies and the growth challenges of *M*. *stenopetala*. As a result of these efforts, its productivity is being maintained and improved. Furthermore, ethnopharmacological studies have documented indigenous knowledge and are crucial for conserving and utilising biological resources [14]. As a result, preserving indigenous knowledge about the plant’s therapeutic properties is critical for national policies ensuring food and health security. Keeping all these aspects in view, we have undertaken this study to assess the nutraceutical uses of *M*. *stenopetala* and its growth cahllenges among traditional healers in southern Ethiopia.

## Materials and methods

### Description of the study area

**Fig 1:**
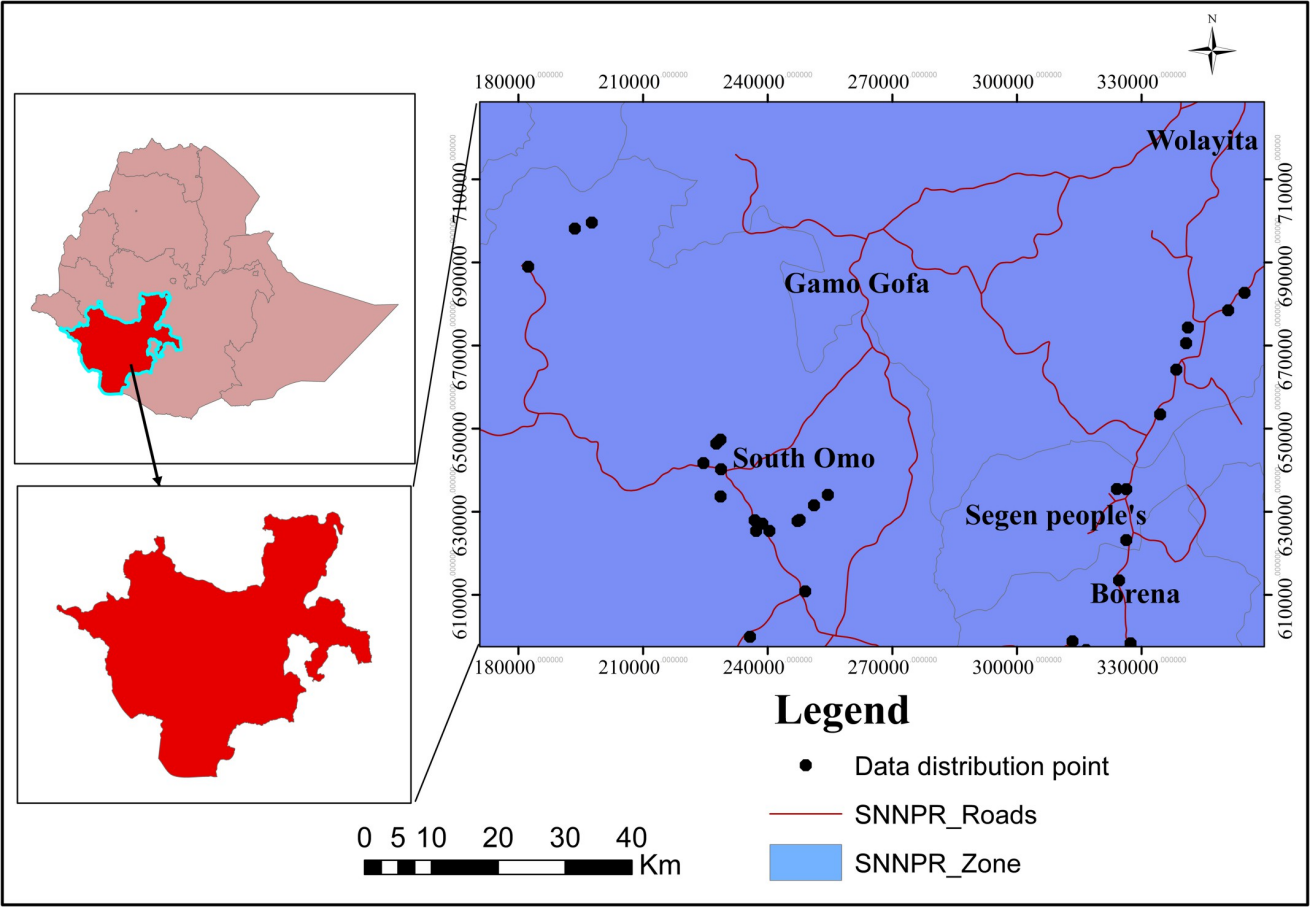
Description of the study area, specific locations of each site in Gamo Gofa, Segen people’s area, and south Omo zones, Ethiopia.

The study was carried out in 50 selected kebeles (the smallest administrative unit) in Gamo Gofa, South Omo and Segen areas of Southern Nation Nationalities and peoples regional state, Ethiopia, from May to June 2020. The region is bordered to the south by Kenya, the Ilemi Triangle to the southwest, Sudan to the west, the Gambela region to the north, and Oromia to the north and east. According to the central statistical agency report, the area is predicted to have a total population of 14,901,990, with 7,408,993 men and 7,492,997 women and the majority of them ie.,13,625,000 (91.4%) were living in rural areas and 1,277,000 in urban areas. (8.57%) [15]. The region consisted of eleven zones and seven distinct woredas. However, among these, Gamo Gofa, south Omo, and Segen zones are the potential hotspot growth areas of *M*. *stenopetala* and these places are thought to be "centre of origin" in which the plant was spread to other areas of the country from these sites [5, 6]. As a result, based on some criteria, 50 kebeles were purposefully selected by consultation with elders and local authorities. These are i) residents of traditional healers, ii) the availability of *M*. *stenopetala* and iii) experience in using it. Each study area was presented **(Fig 1**), which had been sketched using the Ethiopia administrative shape files [16] and map data (**S1 Table**).

### Data collection and procedure

In this study, a total of 192 answerers (89 men and 31 women) were recruited. Out of these, 89 (74.1) were traditional healers (experienced in using Moringa as traditional herbal medicine and had a history of using the plant to treat any aliments), and 25.9% of them were selected from the general population (lay) using a simple random sampling technique. A brief group discussion with key informants was made at each kebele prior to before data collection on nutritional, therapeutic uses and growth challenges of *M*. *stenopetala* [7–9]. Face-to-face interviews and direct field observation with semi-structured questionnaires were used for collecting quantitative data.

### Data quality assurance

Before the study, the questionnaire was pre-tested in 5% of the sampled study population in the Wolayta Zone. A change was made as needed to improve consistency, coherence, and terminology. In addition, the data collectors were trained to complete questionnaires, conduct observations, and manage group discussions with key informants. The data collectors thoroughly checked the completeness of the questionnaire and the saturation of ideas from the group discussion during the data collection phase. Throughout the data collection period, the principal investigator and supervisors provided proper monitoring. At the end of each data collection day, supervisors were also required to cross-check for accuracy and consistency of data, and corrective discussions were held with all research team members as needed. The interviews were conducted in the " regional language, Amharic ". Data were collected two or three times during the study period to confirm the reliability of the ethnopharmacological information. Controversial responses were rejected accordingly.

### Data analysis

#### Quantitative data analysis

The data obtained from the survey were analysed using descriptive and quantitative statistical methods. The pharmacological data were analysed using frequency and fidelity level (FL). The FL was expressed in terms of therapeutic claims using the following formula [17].

$$\text{FL}=\frac{\text{Mx}}{\text{Mt}}\times 100$$

Where Mx is the number of informants who mentioned a specific method of use (recipe) for a given disease and Mt is the total number of members (informants) who mentioned any specific manners of use.

#### Qualitative data analysis

Qualitative data were analysed based on a thematic framework using Open code (version 4.03). Data obtained from focus group discussions and interviews were accurately transcribed, and each data set was coded based on similarity. The key informant interviews were transcribed verbatim by a third party. The transcripts were then reviewed by the first author and compared with the audio recordings and then confirmed by the second author. Corrections were made to ensure the accuracy of the transcription. Data were analysed using the six steps of thematic analysis [18]. These are familiarising with the data, generating initial codes, searching for themes, reviewing the themes, defining and naming the themes, and producing the report. Finally, familiarisation was achieved by listening to the audio recordings, checking that the transcription was accurate, and reading through the transcribed scrips several times before coding. The data were then assigned initial codes while keeping an open stance to allow for modification of codes as the analysis proceeded. Categories were developed from the codes that had a relationship with each other and these were then built into themes. All authors were involved in the process of qualitative data analysis to enhance validity. The four criteria for the trustworthiness of qualitative data were adhered to. Credibility and dependability have been assured through triangulation; transferability has been enhanced by the thick description in the key informant narratives and confirmability was enhanced through supervisory oversight by the third author.

### Ethics approval and consent to participate

Ethical clearance was obtained from the Institutional Review Board (IRB) of Arba Minch University, Arba Minch, Ethiopia. Before the commencement of the study, all participants gave their informed consent. All parties retain their rights to use and authorship of all traditional knowledge. Any use of this material, except scientific publications, requires prior authorisation from the traditional owners and agreement on access to the benefits derived from continuous use.

## Results

### Socio-demographic characteristics

Out of the total 120 study participants, the majority of them were male, with a mean age was 49.10(±6.37 years). It was found that 73.3% of them were illiterate, and more than eighty percent of the respondents were of the Gamo Gofa ethnicity. About 70% of the respondents were farmers (Table 1).

**Table 1:** Socio-demographic characteristics of the participants, May-June 2020.

Variables		Frequency	Percent
Sex	Male	89	74.1
	Female	31	25.9
Occupation	Farmer	84	70.0
	NGO	24	20.0
	Employer	11	9.2
	Merchant	1	0.8
Resident	Gamo Gofa	97	80.8
	South Omo	20	16.7
	Segen	3	2.5
Educational Status	Illiterate	88	73.3
	Primary	24	20.0
	Secondary	8	6.7

### *Moringa stenopetala*: Usage experiences, challenge and knowledge transmission

Qualitative data showed that the *M*. *stenopetala* is a natural gift with tremendous value in the daily life of the community in the study area. Interestingly, two genotypes of *M*. *stenopetala* with different vernacular names were distributed in the study area. To start with, genotype is characterised morphologically by long, thin, smooth, and deep green leaves with purple leaf petioles and a beautiful bunch. It cooks quickly and has a pleasant flavour. It is drought tolerant and has large leaves than its counterpart. This genotype is known as woman moringa "**Setie haleko**" in Gamo zone around Arba Minch, Desert Moringa "**yebereha/yeqola haleko**" in Knoso and the vicinity, whereas Goat moringa "**Yefiyel haleko**" in south Omo around south Ari. The second genotype is known as man Moringa "**Wondie haleko**" in Gamo zone around Arba Minch, highland moringa "**Yedega haleko**" in Konso and the vicinity, bovine moringa "**Yekebtoch haleko**" in South Omo around South Ari, which has short, wide, rough leaves with light green color and there is no formation of purple colour on the leaf petioles. It is unfit for human consumption due to its bitter flavour and lengthy cooking time.

The findings revealed that traditional healers faced a variety of challenges while participating in the preparation of herbal medicine as well as treatment advice and process. These include, i) some members of the community are unwilling to use herbal medicine because they wrongly believe it is illegal and they are afraid of the side effects and, ii) demoralised traditional healers tagged with derogatory names such as ’**Kitel Betash**’, ’**Sirmash**’, **’Asmategna’**, and ’**Metetegna**’ which emotionally traumatised their traditional healing procedures in the community. This has an impact on the social lives of the traditional healers and may lead to isolation from the community. In addition, the report confirmed the absence of written documentation on herbal medicine preparation guides and their use accessed by traditional healers. However, they passed on the information to the next generation secretly through word of mouth (orally), and some of the indigenous knowledge was lost over time due to the difficulty of memorising it.

### Biopotentials of *Moringa stenopetala*

The result anticipated that the plant has various values in their respective community. The majority of the respondent claimed that it is used in traditional foods as a leafy vegetable. It was found from our survey that fresh leaves are garnered from matured plants and subjected to a drying and sieving process. It is then used as an ingredient in traditional dishes such as **Kurkufa, Fosessie** and **Shikerker**. Notably, the mode of preparation and use can range from raw eating to syrup preparation. Interestingly, respondents claimed that they took moringa syrup to alleviate their hunger and tiredness. Furthermore, moringa leaf powder significantly improves maternal and child health by providing an adequate amount of iron and other essential nutrients to the mother. These findings were in line with the previous report that six spoonfuls of leaf powder of *M*. *oleifera* will satisfy nearly all of a woman’s daily iron and calcium needs during pregnancy and breastfeeding [19, 20].

**Fig 2:**
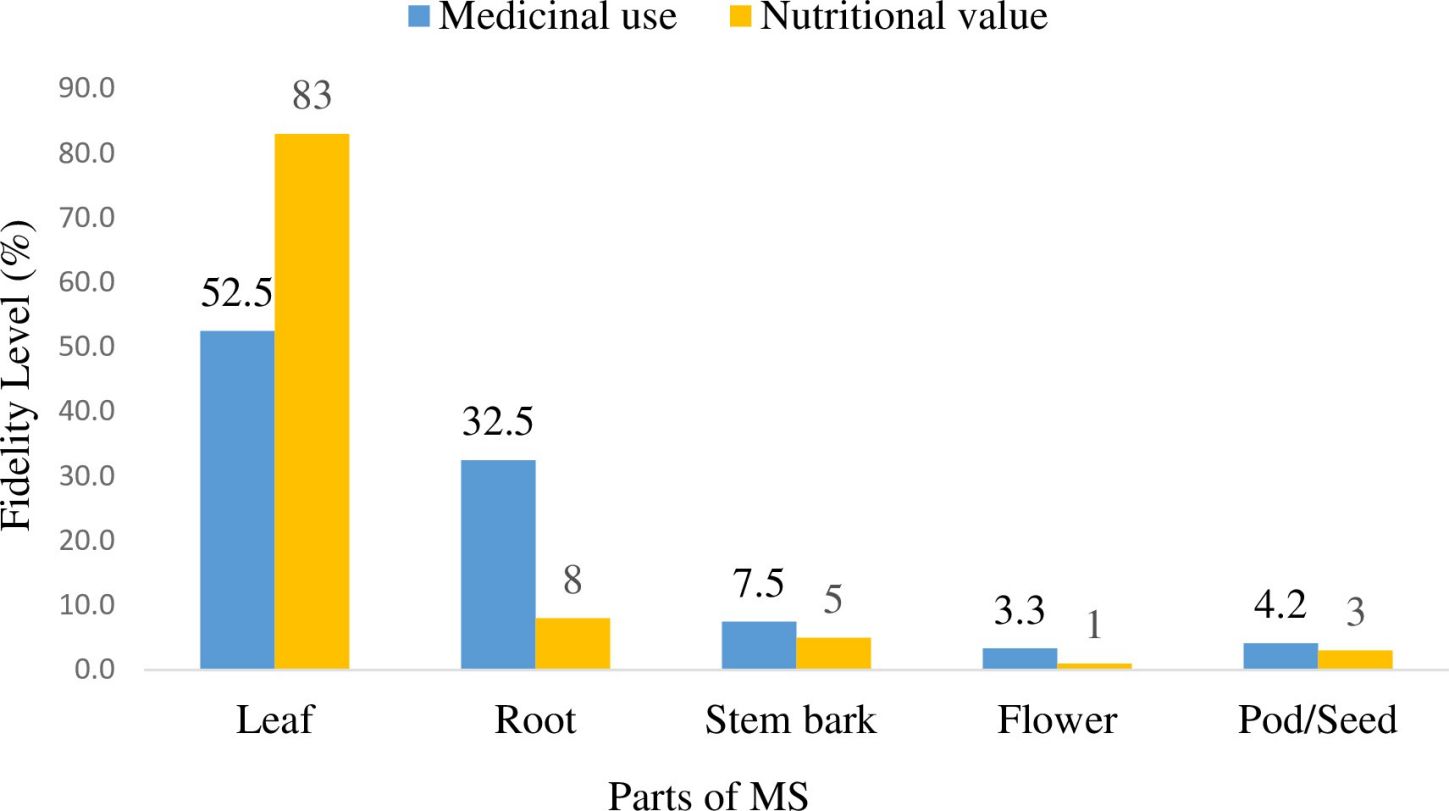
Preference index of *M*. *stenopetala* for nutraceutical values among traditional healers in southern Ethiopia, 2020.

It was found from the result that every part of *M*. *stenopetala* is utilised in the preparation of daily meals and food ingredients. The FL, on the other hand, confirmed that the leaf was high in nutritional values with a different mode of utilisation (Fig 2).

### Therapeutics uses of *M*. *stenopetala*

According to our findings, the majority of study participants rely on *M*. *stenopetala* to treat simple to complex ailments in a variety of ways. Their mode of preparation and utilisation varies according to the plant parts and the types of disease targeted. Our most intriguing finding is that every part has the potential to treat various ailments with different preferential indexes. The leaf (52.5%) is the most commonly used plant part, followed by the root (32.5%) and stem bark (7.5%). The flower, on the other hand, is used to treat rectal dysfunction with a low fidelity level. The variation in preference could be due to the lack of knowledge for those who ignore the specific part, as the information is transferred secretly. The leaf/root was used to treat common colds, malaria, asthma, high blood pressure, diabetes mellitus, heart disease, suppression of kidney stone formation, and uterine rejuvenation through placenta shedding.

In addition, Furthermore, root smoke has been used to treat epilepsy (Table 2). On the other hand, findings of the qualitative data revealed that the leaf, root, stem bark, pod, seed and flower are used to treat various human ailments. As per the indigenous knowledge of its medicinal uses, the leaves were used to treat malaria, high blood pressure, asthma, diabetes, and anaemia, as well as bleeding, wounds, skin drying, diarrhoea, and ear and eye infections. It also improves bone and dental health, regulates blood sugar and fat levels, aids in weight loss, and keeps the liver and kidney health. One of the most significant findings in the report concerned roots. The root smoke is used to treat epilepsy, sexual dysfunction, kidney stone, heart disease and vascular conditions. It also aids the removal of undesirable uterine waste, increases appetite, and reduces hypertension by increasing urine.

**Table 2:** Therapeutics uses and modes of delivery of *M*. *stenopetala* among traditional healers of southern Ethiopia from May to June 2020.

Illnesses treated	Recipes	Guidelines for use	FL%
Leaf			
Headaches	Powder of leaves	Orally, chopping with water, Mixed with tea drink	100
Anaemia and malaria	Powder of leaves	Make the syrup and consume it in the morning	100
Bleeding	Leaf powder	Smearing	33
Cough	Leaf powder	Drink regularly	91.6
Pain and diarrhoea	Leaf powder and root	Drink regularly	96
Inflammation of the ears and eyes	Leaf powder	Grind fresh, carefully washed leaves; squeeze out the juice, and put a few drops in your eyes and ears	37.5
For bone and tooth health	Leaf powder	Orally, crush with water and combine with tea	16.7
For weight loss and to fight bacteria	Leaf powder		83.3
For the health and strength of the skin	Powder of leaves	Orally, crush with water and combine with tea.	93.3
STI	Fresh leaves + 7 small hot peppers + salt + wine combined in a bottle	Drink one tiny glass of the potion every morning and evening until you have recovered for 15 days.	74.2
To treat high blood pressure	Powder of leaves	Put the food in regularly not too hot	60
To treat Infection	Dried leaves or powder of leaves + traditional alcohol	For 7 10 days, drink one glass in the morning before meals.	79.2
To overcome Infertility	Powder of leaves	Put in the meals (not very hot) until the conception	70.8
To treat intestinal worms	Powder of leaves	Put in meals (not very hot) every day for 7 days	95.83
To treat fever	Grind, boil or soak leaves in water	Bathe with the potion	37.5
To treat stomach pains	Powder of leaves	Put in meals (not too hot) for 2–3 days	68.3
To treat headache and migraine	Grind fresh, carefully washed leaves; press out the juice	Put some drops on the eyes or massage the forehead in case of pain	79.2
To treat typhoid fever	Infusion of leaf in association with other leaves	Drink regularly for 7–10 days	62.5
To treat flu and sinusitis	Grind fresh, carefully washed leaves; press out the juice	Put some drops of the juice in your nostrils in case of attack	56.7
Roots			
To increase appetite	Crushed root + lemon	Drink a glass of it before a meal	100
To treat dysentery	Crush the fresh root and press out the juice	Drink 2 teaspoons/day for 5–7 days	100
To treat diseases of the heart and blood vessels	Crushed the root and prepare the syrup	Drink a glass of syrup /day for one month	85
To treat Stomach pain	Powder of dried roots + water alcohol or crushed fresh roots + alcohol	Drink 3 small glasses/day morning, afternoon, and evening until well	86.7
To treat Swellings	Crush fresh root	Apply the paste to the swelling morning and evening until well	84.2
To prevent Tooth decay	Wash, scratch the root, and cut it in small pieces	Place a piece on the rotting tooth at bedtime till it is well.	90
Headaches and migraine	Crush the fresh root and press out the juice	In the event of an attack, put some drops in your eyes and massage your forehead with them.	80
Flu and the common cold	Scrape root and put in a handkerchief	Inhale the smell in case of an attack	72
To prevent the formation of kidney stones	Crushed root + water	crush the roots and use a mixture of salt	86.4
Bark			
Tooth decay	Cut bark as a chew stick	Morning and evening, chew on a chew stick and hold the juice in your mouth for a few moments until it is well absorbed.	72
For acne and gout	Cut bark as a chew stick	Morning and evening, chew on a chew stick and hold the juice in your mouth for a few moments until it is well absorbed.	
Fever	Soak bark in water	Use water for a bath for 3–5 days	86
Stomach pains	Cut bark as a chew stick	Chew on a chew stick and swallow the juice at will	67
Remove unwanted remnants of the uterus.	Cut the bark, crush, and mix the powder with water.	Boiled with water and drink after cool for 1 month	
Malaria	Soak bark, collect the mousse, and add sugar	Drink 2 small glasses/day for 5–7 days	96
Indigestion	Cut bark as a chew stick	Morning, midday, and evening, chew on the chew stick and consume the juice till well.	83
Malaria	We carefully dry the washed pods without seed and soak it in water	Drink 3 glasses/day morning, afternoon, and evening 5–7 days	86
Cure liver and pancreatitis	Collect the pod and pod bark carefully and dry, then powder and mix with boiled water	Drink the syrup in the morning before the meal for 3 months before a meal	83
To cure acne and gout	Collect the pod and pod bark carefully and dry carefully, then powdered	For at least one month before a meal, drink the syrup in the morning	96
Fever	Soak pod in water	Bathe with it for 3–5 days	100
Seeds			
Diabetes	Remove the winged shell of the seed	Eat 2 kernels a day regularly	86
Gout and Epilepsy	Remove the winged cover of the seed.	Smoking	73
Sexually transmitted diseases, urinary tract infections	Remove the winged shell of the seed	Eat five kernels in the morning, afternoon, and evening at will	96
Sexual weakness	Remove the winged shell of the seed	Eat two kernels in the morning, afternoon, and evening at will	80
Flower			
Increases milk production for breastfeeding mothers	Pounding & mixing with water	Drink regularly	96.7
For kidney and urinary tract infections as well as bladder infections	Pounding & Mixing	Drink regularly	91.2
Stimulates liver function	Pounding & Mixing	Drink regularly	90

Furthermore, the root crushed mixed with salt can be used to treat gout (**hyperuricemia)**. Ethnopharmacological data showed that the seeds are used to treat urinary tract infections, gout (hyperuricemia), epilepsy, and sexually transmitted diseases. The flowers, on the other hand, are used to treat kidney, urinary tract, bladder infections, sexual dysfunction, liver disease, pancreas infections and intestinal worms. Abnormal body system processes that result from malnutrition can all be treated with the use of pods and barks (Table 2).

### Parts of *M*. *stenopetala* and preparation of the remedy

**Fig 3:**
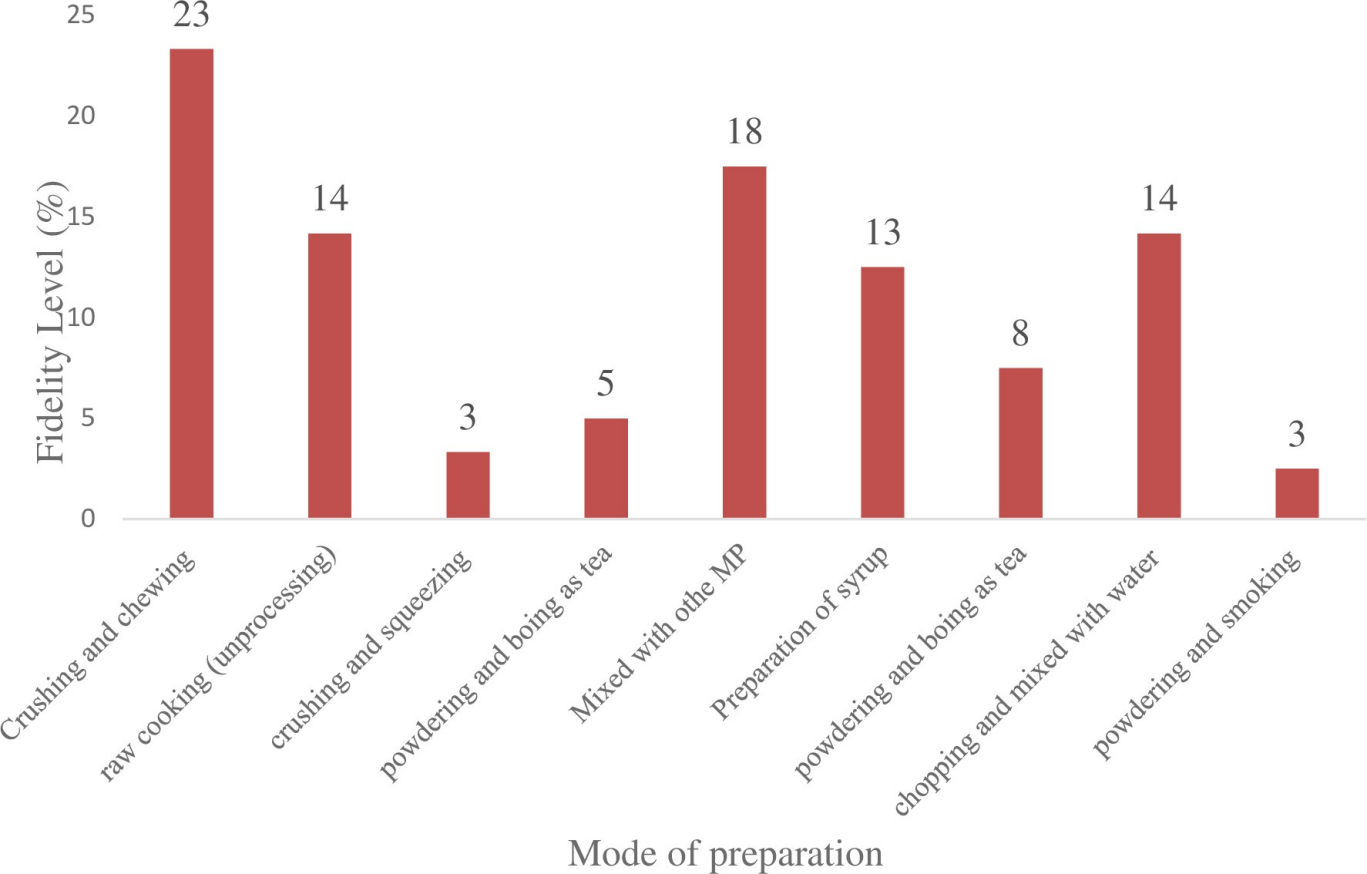
Method preference for the preparation of remedy from *M*. *stenopetala*.

The data revealed that different preparation methods were applied to formulate the required herbal remedy. Crushing and pulverising fresh, blending with other medicinal plants, and raw cooking of plant parts were the significant methods of preparation of therapeutic formulations, which account for FL of 25, 18 and 14%, respectively (Fig 3).

### Methods of treatment

A wide range of traditional treatment applications is included in this investigation. However, the most common means of application were drinking/eating/chopping (24.2%), chewing (22.5%) and spreading on affected portions of the body/skin (19.2%), respectively. On the other hand, wrapping and dropping on the affected areas were rarely used during treatment (Table 3).

**Table 3:** Methods of application of *M*. *stenopetala* herbal remedy for treating human ailments among traditional healers of southern Ethiopia, 2020.

Methods of applications	Frequency	Percentage
Drinking/eating/chopping	33	27.5
Chewing	29	24.2
Smearing/wrapping	23	19.2
Inhaling	9	7.5
Dropping on affecting areas	7	5.8
Washing	10	8.3
Holding on teeth and spitting out	9	7.5

### Dosage and routes of administration

**Fig 4:**
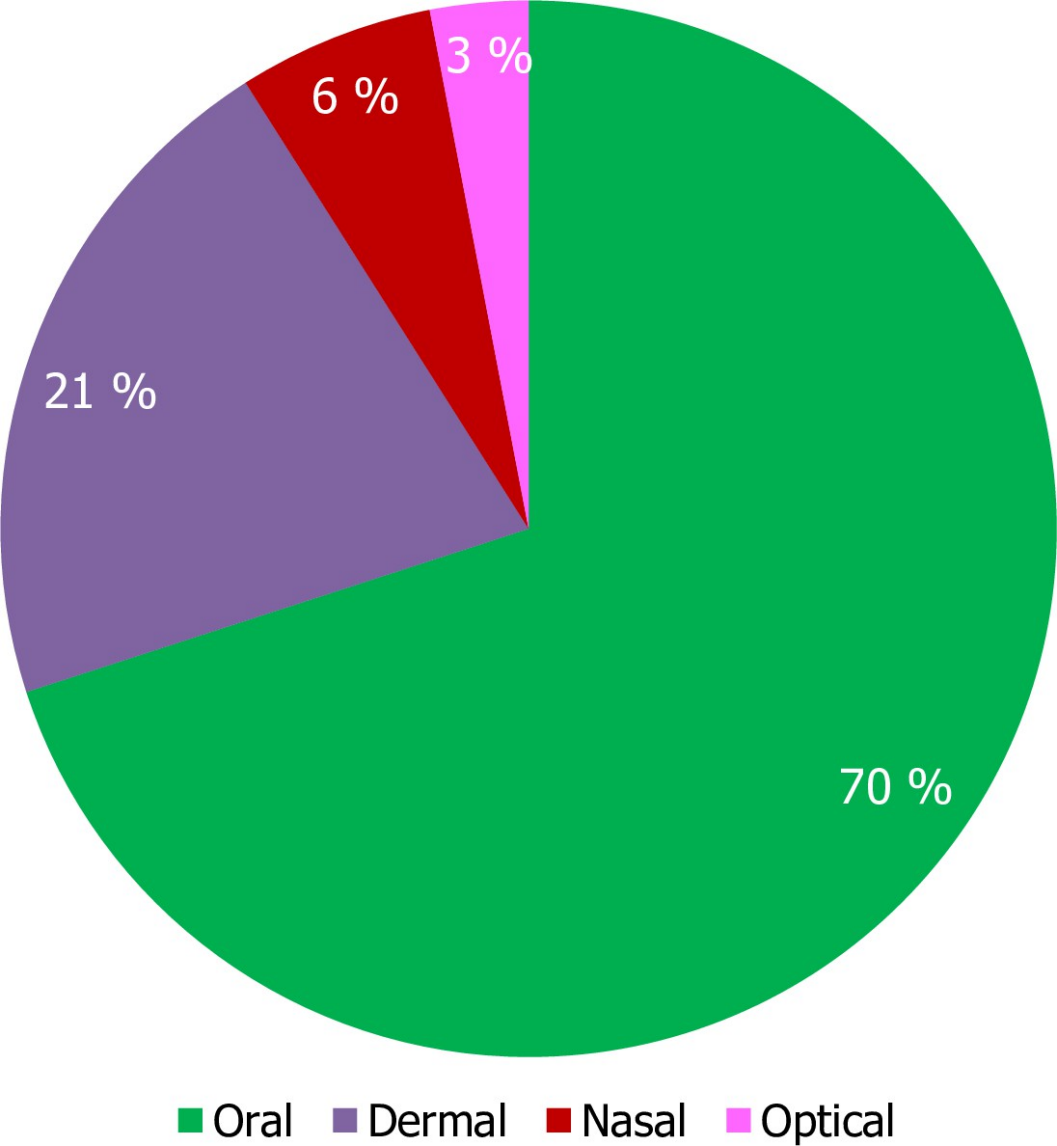
Mode of the remedy administration prepared from different parts of *M*. *stenopetala* in southern Ethiopia, May- June 2020.

Oral intake is the primary route of administration (85%), followed by dermal application (71%), while the ocular canal (25%) is the least used organ (21%). However, nasal (6%) and ocular (3%) delivery methods were rarely used depending on plant parts and types of aliments. The dosage of the crude drug is determined by the patient’s age, sex, physical condition, and the severity of the disease; however, the methods used are not precise enough to establish the exact dosage. Measurements such as finger length for bark, root, and stem; pinch for powdered plant material, various measuring devices including teaspoon, coffee cup, teacup, and glass cups, and the number of extract drops, leaves, seeds, seeds per pod, roots, and flowers are also in use (Fig 4).

### Ecological services and production constraints of *M*. *stnopetala*

The study confirmed that the major ecological functions of the plant include preventing soil erosion (46.7), and providing shelter for animals and humans during the dry season (40.0%). These findings were consistent with the result of qualitative studies that revealed that Moringa is utilised as fire fuel, fencing, and water purification. Besides, it is also used as a natural insecticide, gum, honey clarifier, fertiliser, paper pulp, rope, and rope-making material. Moringa is also used as a live fence and decorative plant in its natural habitat. Moringa has no allelopathic effect and is employed in mixed cultures with other food plants. The extended roots of the moringa plant expand laterally and deeply into the ground, making them crucial for maintaining the soil.

Additionally, it serves as food for bees. The usage of moringa oil as a moisturiser and skin care agent in body and hair care demonstrates its tremendous cosmetic value. Ointments and skin treatments have incorporated moringa oil. However, the findings revealed that the moringa moth (55.8%) and biomass production (required ample space) is the most critical environmental factors threatening productivity and sustainability (Table 4). Qualitative data confirmed that numerous biotic and abiotic factors affect plant productivity. The moringa moth is the most pervasive impediment to plant growth. The most significant growing obstacle is the disease brought on by the pest (Moringa moth). Infestations happened after the wet season as the sun rose in the south in May and June and elsewhere in November and March. Because the plant is sensitive to waterlogging, yellow leaves are a sign of waterlogging. The tree’s output is restricted because it is big and needs additional land. In general, the plant production is low, the region’s natural resources are underutilised, inadequate market access, and a need to improve production and market value chain analysis.

**Table 4:** Ecological service, use, and production constraints of *M*. *stenopetala* from southern Ethiopia.

Variables/parameter		Frequency	Percent
Major production constraints	Moringa moth	67	55.8
	Biomass production (need large space)	40	33.4
	waterlogging	13	10.8
Intercropping crop	Coffee	71	59.2
	Maise	31	25.8
	Teff	18	15.0
Ecological importance	soil erosion protection	56	46.7
	shelter for different animals s in the dry season	48	40.0
	Water and environmental purification	13	10.8
	Ecological balance	3	2.5

## Discussion

This study presents ethnopharmacological data on the *M*. *steneopetala* and growth challenges in southern Ethiopia. Despite their community service, traditional healers have faced numerous challenges, such as a lack of documentation on the use of herbal medicine, how it is prepared, and how it is used because the information is passed on orally and secretly. This finding was consistent with previous research, which found that information about herbal medicine was secretly obtained from their ancestors to maintain its healing potential [21]. On the other hand, traditional healers and members of society reported that traditional medicinal practices are not encouraged by the Kebele government agencies, which are considered illegal activities. The results of qualitative data revealed that the majority of the healers also reported their free practice in traditional medicine was reduced due to experiencing derogatory descriptions and abusive terms calling them witchcrafts, **KITEL BETASH**, **SIRMASH**, **DEBTERA**, and **ASMATEGNA**, as explained by the traditional healers, making them to isolated from the community. These findings were supported by previous research that reported that traditional healers faced many community challenges and reduced their interest in the practice of herbal medicine [21]. Conversely, some traditional healers are not interested in obtaining legal or governmental recognition for their traditional healing practices as the potential of herbal medicine will dwindle during the publicity period.

Results of the study have indicated that *M*. *stenopetala* is still playing a significant role in meeting the daily basic food and healthcare need of the people of southern Ethiopia. It plays an important role in the daily life of the community as a source of nutrition and herbal medicine. The leaf followed by the root was highly accessed for its nutritional values. These findings are consistent with the results of a previous study that confirmed leaf, flowers and pods are used as staple foods [9, 22]. Furthermore, the proximate analysis of moringa leaf confirmed that it has a wealth of essential, disease-preventing constituents, protein, carbohydrates, fibre, vitamins, and minerals such as calcium, iron, potassium, zinc, phosphorus [2] and vitamins such as vitamin C and vitamin A [2, 23]. This elucidates the provided balance diet throughout the year, especially for the pleasant community in which another option is limited, therefore combating malnutrition by providing all essential minerals and nutrients. It is found in the current study that *M*. *stenopetala* has various therapeutic uses with tremendous healing potential against a wider range of human ailments. This result was in line with the findings of previous research that revealed either part of Moringa was used to treat skin infections, depression and anxiety, asthma, blood impurities, cholera, glands swelling, headache, conjunctivitis, cough, diarrhoea, eye and ear infections, fever, abnormal blood pressure, joint pain, respiratory diseases, urinary tract infections [24–26], sore throats, tuberculosis, intestinal infections [1, 6, 9], diabetes, unwanted pregnancy [1], stomach aches, diabetes, and lowers cholesterol levels in the liver [27]. Accordingly, it was found that the leaf followed by the root and the bark is a common practice in many communities in the study area, as reported in Ethiopia [21, 28], Bolivia [29], and Kenya [30]. The high utilisation rates of leaves could be attributed to the ease with which they can be obtained in large quantities compared to other plant parts. Leaves are the main photosynthetic organ in plants and are considered to be a vital component of the natural pharmacy for the synthesis of constituents, particularly those that are more pharmacologically active against diseases [31]. Moreover, roots were the common plant parts used in herbal medicine preparations, and the other parts were underutilised. However, a clear relationship exists between the parts of the plant collected or the collection method and the impact on the harvested plant [32]. Thus, utilisation of the bark and root is damaging and makes moringa genotypes vulnerable to overexploitation.

The findings confirmed that various methods of preparation and utilisation were practised. However, crushing is most commonly used to prepare herbal medicine from different parts of *M*. *stenopetala*. A similar result was obtained from previous research studies that reported that [33–35] crushing is the preferred way of preparation. The study indicated that the oral route was the most common way of administration of herbal medicinal products in the study area. This was supported by [36]. most of the orally administered remedies showed a higher prevalence of internal disorders in the study area. However, the dose in the oral system should be administered with more caution than in the dermal form, as it could cause other serious internal problems. Similarly, various research results have mentioned oral use as the primary route of administration in traditional herbal medicines [37, 38].

Despite its nutraceutical values, *M*. *stenopetala* has ecological services such as protection from soil erosion, water purification, intercropping and sources of fire food and many cosmetic products [11]. It protects against soil erosion [39], provides shade and balances the ecology [1]. The productivity of *M*. *stenopetala* is hampered by biotic and abiotic factors. Among this attack with moringa moth (*Noorda blitealis*) is the predominant growth challenge. This pest occurred in May, June, November and March after the sun rose, followed raining. This result was in agreement with previous research that reported that the productivity of Moringa was challenged by the moringa moth [40, 41].

## Conclusions

*Moringa stenopetala* has remarkable nutraceutical applications and ecological services which play a significant role in the daily life of a community in southern Ethiopia. Different approaches were employed to prepare daily meals and remedies from the plant. Interestingly, the potential of this plant in treating epilepsy, leprosy, and hepatitis/liver disease was recorded in this document. Therefore, it’s necessary to conduct further experimental pharmacological research to unravel the hidden potential of the plant to support a milestone in drug discovery. Moreover, attack by the moringa moth is one of the major growth challenges in the study area, resulting in low production of the plant.

### Limitation of the study

The study has a smaller sample size, and also, the majority of therapeutic uses surveys are self-reported by respondents and unconfirmed by pharmacological investigation.

## Supporting information

S1 File Ethiopia’s administrative shape files used for creating the study map. (MXD)

S1 Table Geographical coordination/map data/ of each site included in the study. (DOCX)

S1 Data (DOCX)
