# Supplementary material for: Ethno-pharmacological investigations of Moringa stenopetala Bak. Cuf. and its production challenges in southern Ethiopia
Source: PLoS One. 2022 Sep 23;17(9):e0274678. doi: 10.1371/journal.pone.0274678 (PMC9506611; doi:10.1371/journal.pone.0274678)
Supplement: S1 Table — (DOCX) [file pone.0274678.s002.docx]

Supplementary table 1: Geographical coordination/map data/ of each site included in the study.

| Location name | Latitude | Longitude | Altitude (m) |
| --- | --- | --- | --- |
| Arkisha | 6.35468 | 37.0324075 | 1435 |
| Keyssa | 6.25387 | 37.0237243 | 1423 |
| Kako | 6.25387 | 37.0240419 | 1412 |
| Algakebelle | 6.40214 | 37.0228778 | 1427 |
| Geribol | 6.27763 | 37.0247201 | 1337 |
| Tlinde | 6.31556 | 37.0251160 | 1338 |
| Aluma | 6.34066 | 37.0254501 | 1247 |
| Chali | 6.28034 | 37.0247714 | 1333 |
| KeyAfer | 6.10848 | 37.0249056 | 1608 |
| Alduba | 5.99920 | 37.0235763 | 1330 |
| Kako | 6.27126 | 37.0238738 | 1345 |
| Goldia | 6.27951 | 37.0236894 | 1394 |
| Hana | 688982 | 37.0182362 | 585.2 |
| Giyo mender 3 | 698146 | 37.0193602 | 711 |
| Giyo men(shumuto) | 6.99587 | 37.0197680 | 795.9 |
| Tenadam sefer | 461640 | 37.0229663 | 1432 |
| Giza | 6.46411 | 37.0227645 | 1388 |
| Aykamer | 6.47361 | 37.0228652 | 1439 |
| Kuri | 6.41702 | 37.0224541 | 1328 |
| Betsemal | 6.33679 | 37.0228669 | 1380 |
| Woito | 5.977681 | 37.0277662 | 575.7 |
| Ligno/Kekerti | 5.977681 | 37.0296032 | 1321 |
| Kashawoto/Arfaide | 5.98828 | 37.0313391 | 1560 |
| Lehaytie (madria) | 5.96724 | 37.0316681 | 1475 |
| Gera | 5.83721 | 37.0322271 | 1649 |
| Gayle | 5.98348 | 37.0327348 | 1178 |
| Gato | 6.13474 | 37.0324594 | 1273 |
| Chralie | 6.23185 | 37.0326326 | 1999 |
| Zeyssie | 6.35425 | 37.0326326 | 1170 |
| Selesira | 6.53449 | 37.0334473 | 1116 |
| Molle | 6.92547 | 37.036778 | 1218 |
| M/abaya | 6.95696 | 37.0363725 | 1223 |
| Wajifo | 7.13077 | 37.0361360 | 1224 |
| Kola barena | 7.11449 | 37.0361203 | 1250 |
| Fura | 6.82703 | 37.0354795 | 1196 |
| Lante | 6.78489 | 37.0350863 | 1194 |
| Chano mille | 6.76165 | 37.0344661 | 1215 |
| Shara | 6.74333 | 37.0341154 | 1257 |
| Arbaminch/Secha/ | 6.64210 | 37.0338383 | 1411 |
| AMU | 6.70567 | 37.0340749 | 1217 |
