# Supplementary material for: Ethno-pharmacological investigations of Moringa stenopetala Bak. Cuf. and its production challenges in southern Ethiopia
Source: PLoS One. 2022 Sep 23;17(9):e0274678. doi: 10.1371/journal.pone.0274678 (PMC9506611; doi:10.1371/journal.pone.0274678)
Supplement: S1 Data — (DOCX) [file pone.0274678.s003.docx]

| **CS** | **ELEVATION** | **LONGITUDE** | **LATITUDE** |
| --- | --- | --- | --- |
| **Arkisha** | 1435 | 37N^0^0324075 | 0635468 |
| **Keyssa** | 1423 | 37N^0^ 0237243 | 0625387 |
| **Kako** | 1412 | 37N^0^ 0240419 | 0625387 |
| **Algakebelle** | 1427 | 37N^0^0228778 | 0640214 |
| **Geribol** | 1337 | 37N^0^0247201 | 0627763 |
| **Tlinde** | 1338 | 37N^0^0251160 | 0631556 |
| **Aluma** | 1247 | 37N^0^0254501 | 0634066 |
| **Chali** | 1333 | 37N^0^0247714 | 0628034 |
| **KeyAfer** | 1608 | 37N^0^0249056 | 0610848 |
| **Alduba** | 1330 | 37N^0^0235763 | 0599920 |
| **Kako** | 1345 | 37N^0^0238738 | 0627126 |
| **Goldia** | 1394 | 37N^0^0236894 | 0627951 |
| **Hana** | 585.2 | 37N^0^0182362 | 0688982 |
| **Giyo mender 3** | 711 | 37N^0^0193602 | 0698146 |
| **Giyo men(shumuto)** | 795.9 | 37N^0^0197680 | 0699587 |
| **Tenadam sefer** | 1432 | 37N^0^0229663 | 0461640 |
| **Giza** | 1388 | 37N^0^0227645 | 0646411 |
| **Aykamer** | 1439 | 37N^0^0228652 | 0647361 |
| **Kuri** | 1328 | 37N^0^0224541 | 0641702 |
| **Betsemal** | 1380 | 37N^0^0228669 | 0633679 |
| **Woito** | 575.7 | 37N^0^0277662 | 05977681 |
| **Ligno/Kekerti** | 1321 | 37N^0^0296032 | 05977681 |
| **Kashawoto/Arfaide** | 1560 | 37N^0^0313391 | 0598828 |
| **Lehaytie (madria)** | 1475 | 37N^0^0316681 | 0596724 |
| **Gera** | 1649 | 37N^0^ 0322271 | 0583721 |
| **Gayle** | 1178 | 37N^0^ 0327348 | 0598348 |
| **Gato** | 1273 | 37N^0^ 0324594 | 0613474 |
| **Chralie** | 1999 | 37N^0^  0326326 | 0623185 |
| **Zeyssie** | 1170 | 37N^0^ 0326326 | 0635425 |
| **Selesira** | 1116 | 37N^0^ 0334473 | 0653449 |
| **Molle** | 1218 | 37N^0^ 036778 | 0692547 |
| **M/abaya** | 1223 | 37N^0^ 0363725 | 0695696 |
| **Wajifo** | 1224 | 37N^0^ 0361360 | 0713077 |
| **Kolaberena** | 1250 | 37N^0^ 0361203 | 0711449 |
| **Fura** | 1196 | 37N^0^ 0354795 | 0682703 |
| **Lante** | 1194 | 37N^0^ 0350863 | 0678489 |
| **Chenomili** | 1215 | 37N^0^ 0344661 | 0376165 |
| **Shara** | 1257 | 37N^0^ 0341154 | 0674333 |
| **Arbaminch/Secha/** | 1411 | 37N^0^ 0338383 | 0664210 |
| **AMU** | 1217 | 37N^0^ 0340749 | 0670567 |
| **Abaroba/ Konso** | **un** | **un** | un |
| **Kawade/Keple** | **un** | **un** | un |
| **Mare /burche** | **un** | **un** | Un |
| **Kerkerma** | **-** | **-** | - |
| **Gato/derashe** | **-** | **-** | - |
| **Holte/kebele/** | **-** | **-** | - |
| **Chano dorga** | **-** | **-** | - |
| **Kola shara** | **-** | **-** | - |
| **AM(sikela)** | **-** | **-** | - |
